# Comparison of the SARS-CoV-2 spike protein ELISA and the Abbott Architect SARS-CoV-2 IgG nucleocapsid protein assays for detection of antibodies

**DOI:** 10.1371/journal.pone.0255208

**Published:** 2021-07-29

**Authors:** Ashutosh Wadhwa, Sherry Yin, Brandi Freeman, Rebecca B. Hershow, Marie Killerby, Anna R. Yousaf, Sandra Lester, Lisa Mills, Sean A. Buono, Mary Pomeroy, Daniel Owusu, Victoria T. Chu, Jacqueline E. Tate, Sanjib Bhattacharyya, Patricia Hall, Natalie J. Thornburg, Hannah L. Kirking

**Affiliations:** 1 COVID-19 Response Team, CDC, Atlanta, Georgia, United States of America; 2 Laboratory Leadership Service, CDC, Atlanta, Georgia, United States of America; 3 Epidemic Intelligence Service, CDC, Atlanta, Georgia, United States of America; 4 City of Milwaukee Health Department Laboratory, Milwaukee, Wisconsin, United States of America; Waseda University: Waseda Daigaku, JAPAN

## Abstract

Serologic assays developed for SARS-CoV-2 detect different antibody subtypes and are based on different target antigens. Comparison of the performance of a SARS-CoV-2 Spike-Protein ELISA and the nucleocapsid-based Abbott Architect^TM^ SARS-CoV-2 IgG assay indicated that the assays had high concordance, with rare paired discordant tests results.

## Introduction

Severe acute respiratory syndrome coronavirus 2 (SARS-CoV-2) has caused over 108 million cases of coronavirus disease 2019 (COVID-19) and over 2.5 million deaths globally [1]. Many COVID-19 diagnostic tests are now available. These include molecular assays such as real-time reverse transcription polymerase chain reaction (rRT-PCR) and rapid antigen tests which are useful for identifying acute SARS-CoV-2 infections [2]. Serologic assays can detect antibodies that can be produced after infection or vaccination, as well as used to understand the presence of antibodies across a population [3].

Specific serologic assays include enzyme-linked immunosorbent assays (ELISA), chemiluminescent immunoassays, neutralization assays, and lateral flow immunoassays [4]. Currently, the SARS-CoV-2 spike (S) and nucleocapsid (N) proteins are the most commonly used targets in serological assays [5]. Natural infections in adults with SARS-CoV-2 should induce antibodies both to the S and N proteins [6]. However, antibody responses to vaccination depend on the antigen proteins within (or induced by) a vaccine formulation. Different assays may also have variable limits of detection and may detect different antibody isotypes (e.g., Immunoglobulin (Ig) A or IgA, IgM, IgG) which can rise at different rates following infection. However, knowledge on SARS-CoV-2 antibody kinetics is limited, which has challenged optimization of test usage and interpretation [7].

To understand how the performance of antibody serologic assays may differ, we evaluated antibody responses in SARS-CoV-2 cases and their household contacts using two assays: the SARS-CoV-2 S-Protein IgG ELISA (spike ELISA) [8] and the Abbott Architect^TM^ SARS-CoV-2 IgG assay (Architect) [9]. Additionally, we investigated whether patient characteristics were associated with differences in assay results.

## Materials and methods

### Study population

A household transmission investigation was performed in Milwaukee, WI in April–March 2020 as previously described [10]. Laboratory-confirmed COVID-19 patients (index cases) and their household contacts were enrolled during March 22–April 25, 2020. Demographic characteristics, medical history, recent symptoms and dates of onset, prior SARS-CoV-2 test dates and results, and household-level information were collected from all participants at enrollment. Two household visits were conducted: one immediately after enrollment (visit-1) and another 14 days later (visit-2). At both visits, nasopharyngeal (NP) swabs and blood were collected from all participants.

### Samples and laboratory testing

NP swabs were tested for the presence of SARS-CoV-2 nucleic acid using the CDC rRT-PCR assay at the City of Milwaukee Health Department Laboratory [11]. All serum samples were tested by the spike ELISA and Architect. The spike ELISA (performed at CDC laboratories) detects all immunoglobulins (pan-IgG), but for the purpose of this comparison only IgG against SARS-CoV-2 spike (S) protein was used [8]. For the spike ELISA, specimens were considered reactive with an optical density (OD) ≥0.4 at a serum dilution of 1:100. As measures of IgG, signal to threshold (S/T) values were calculated by dividing the OD for 1:100 dilution by 0.4. S/T values greater than or equal to 1 were considered positive. The Architect assay (performed at Milwaukee Health Department Laboratories) is a qualitative test that detects IgG antibodies against SARS-CoV-2 nucleocapsid (N) protein [9, 12]. The system calculates a calibrator mean chemiluminescent signal and the default result unit is index (S/C). Index values greater than 1.4 for the Architect assay were considered positive.

### Data analysis

Qualitative results (positive/negative for the presence or absence of IgG; if the IgG signal was above/below the set threshold) as well as the S/T (for spike ELISA) and index values (Architect) were used for serologic assay comparison. All serum samples were categorized as concordant positive, concordant negative, or discordant based on qualitative results from each assay. Kappa statistic was calculated as a measure of agreement between the two assays.

Age, gender, race, ethnicity, underlying medical conditions, reported symptoms, days from symptom onset, rRT-PCR test result before or at the time of serologic testing and days from rRT-PCR positivity to serologic testing were compared among concordant positive and discordant samples in order to understand factors associated with assay concordance. Fisher’s exact test of independence was used to assess if the proportion of concordant positive and discordant samples significantly differed across demographic, clinical, or symptom variables as determined by a p-value less than 0.05. All statistical analyses were performed using SAS 9.4 (Cary, NC, USA) software.

### Ethical consideration

This investigation was part of the ongoing public health response to COVID-19; thus, CDC’s Human Research Protection Office determined the activity to meet the requirements of public health surveillance as defined in 45 CFR 46.102(l)(2) and exempt from human subjects research regulations [13].

## Results

Ninety participants including 26 index cases and 64 contacts from 26 households were enrolled. One hundred five serum samples were collected from 73 unique individuals across 23 households, including 23 index cases and 50 contacts. Fifty-six of 105 serum samples were collected at visit 1, while 49 were collected at visit 2 (i.e., 14 days later). Of all serum samples, 40 (38.1%) were concordant positive, 58 (55.2%) were concordant negative, and seven (6.7%) were discordant. The seven discordant serum samples were from unique individuals and all seven tested positive by spike ELISA and negative by Architect. The overall kappa coefficient value for all serum samples was 0.86, suggesting strong agreement between the two assays.

Table 1 presents demographic, clinical, and laboratory characteristics of the 47 individuals who had positive serology results for one or both assays. Among the 47 respiratory samples collected from these individuals before or during the study period, 43/47 (91.4%) were from individuals who tested positive by rRT-PCR at or before the time of serum collection. Of these 37 (86%) had a positive rRT-PCR result more than 7 days prior to serum collection, while six (14%) were rRT-PCR-positive within 7 days of serum collection. Fisher’s exact test showed that none of the clinical variables, including time from symptom onset, were statistically significantly different between concordant positive and discordant samples.

**Table 1:** Demographic, clinical, and laboratory characteristics of the 47 serum samples tested by both SARS-CoV-2 spike protein ELISA (spike ELISA) and Abbott Architect^TM^ IgG assay (Architect) with at least one positive serology assay result.

	Concordant positive	Discordant serum	P-value¥
	N = 40 (%)	N = 7 (%)	
**Visit number**^α^			0.69
Visit 1	14 (35)	3 (43)	
Visit 2	26 (65)	4 (57)	
**Age (years)**			0.56
0–18	6 (15)	2 (29)	
19–50	22 (55)	4 (57)	
51–90	12 (30)	1 (14)	
**Sex**			0.24
Male	18 (45)	5 (71)	
Female	22 (55)	2 (29)	
**Days since symptom onset**‡*			0.63
0–9	3 (8)	1 (14)	
10–28	23 (59)	5 (71)	
29–44	12 (31)	1 (14)	
Asymptomatic	1 (3)	0 (0)	
**Any positive rRT-PCR test during study period (from pre-enrollment to post-closeout)**			0.49
Yes	37 (93)	6 (86)	
No	3 (8)	1 (14)	
**Timing of positive rRT-PCR test results**			0.45
rRT-PCR positive >7 days prior to serum sample collection	31 (78)	6 (86)	
rRT-PCR positive ≤7 days of serum sample collection	6 (15)	0 (0)	
No previous rRT-PCR positive test	3 (8)	1 (14)	

¶Positive on Spike ELISA and negative on Architect assay.

^α^ Visit 1 refers to the visit immediately after enrollment; and, visit 2 is the final visit 14 days after the initial visit.

¥ P-value from Fisher’s exact test.

‡ Symptom onset included onset of any of the following symptoms: cough, shortness of breath, discomfort breathing, fever, myalgia, headache, chills, loss of taste or smell, or sore throat; asymptomatic means the individuals were asymptomatic prior to and throughout the 14-day follow-up period of the study. Day 0 means first day of symptom onset.

*Missing data: Days since symptom onset: N = 1.

A detailed description of the seven individuals with discordant results is presented in Table 2. The age of the individuals with discordant results ranged from 15–54 years and included both male (n = 5) and female (n = 2) persons. The days from symptom onset to time of sample collection for serologic testing ranged from 0–29 days, and one person was asymptomatic. All the seven discordant samples were positive by spike ELISA and negative by Architect. Six of the seven individuals had a positive rRT-PCR test during the investigation and the time between first rRT-PCR positive test and serum collection ranged from 0 to 29 days.

**Table 2:** Detailed description of individuals with discordant serology assay test results (n = 7).

	Individuals with discordant results (n = 7)						
**Characteristics**	A	B	C	D	E	F	G
**Age**	22	20	18	15	54	41	46
**Gender**	F	M	M	M	M	F	M
**Race/Ethnicity**^α^	White	White	Black	Multiracial	White	White	American Indian/Alaska Native
Days since symptom onset to time of serology testing*	Asym.	22	0	20	29	23	21
**rRT-PCR status at time of serology testing (including previous and concurrent testing)**	P	P	N	P	P	P	P
**Days since rRT-PCR testing to time of serology testing**	9	10	ND	14	25	22	20
**Individual reported past SARS-CoV-2 infection**	No	No	No	No	No	No	No
**Underlying medical condition(s)**							
** Chronic lung condition¶**	No	No	Yes	Yes	No	No	No
** Diabetes**	No	No	No	No	No	No	Yes
** Cardiovascular disease¥**	No	No	No	No	No	No	Yes
** Chronic kidney disease**	No	No	No	No	No	No	No
** Immunocompromising condition‡**	No	No	No	No	No	No	No
** Chronic liver disease**	No	No	No	No	No	No	No
** Neurological condition**	No	No	No	No	No	No	No

Abbreviation: Asym. = Asymptomatic; P = Positive by rRT-PCR; N = Negative by rRT-PCR; ND = No Data.

α All the 7 individuals with discordant results were non-Hispanic.

*Symptom onset included onset of any of the following symptoms: cough, shortness of breath, discomfort breathing, fever, myalgia, headache, chills, loss of taste or smell, or sore throat; asymptomatic means the individuals were asymptomatic prior to and throughout the 14-day follow-up period of the study. Day 0 means first day of symptom onset.

¶ Chronic lung condition included COPD, cystic fibrosis, pulmonary fibrosis, and other chronic lung diseases, asthma, tuberculosis.

¥ Cardiovascular disease included heart failure, coronary artery disease, cardiomyopathies, hypertension, or congenital heart disease.

‡ Immunocompromising conditions included solid organ transplant, blood, or bone marrow transplant; immune deficiencies; HIV with a low CD4 cell count or not on HIV treatment; prolonged use of corticosteroids; or use of other immune weakening medicines.

**Fig 1:**
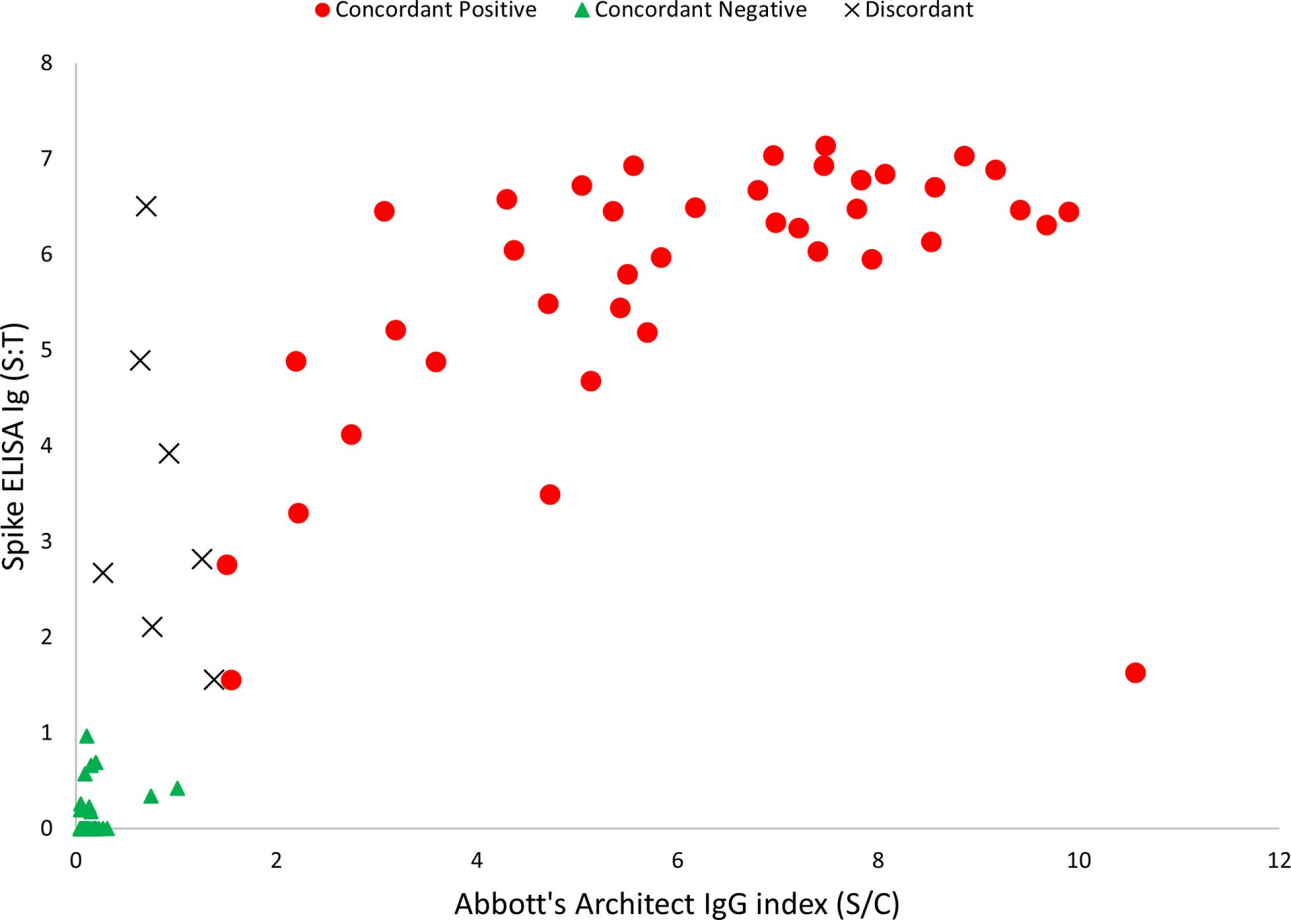
Comparison of the IgG signal detected by the SARS-CoV-2 spike protein ELISA (spike ELISA) and the Abbott Architect^TM^ SARS-CoV-2 IgG assay (Architect) (n = 105 serum samples). IgG signal to threshold (S:T) value only for Spike ELISA and the Abbott Architect^TM^ SARS-CoV-2 IgG (Architect) assay using 105 serum samples. For the Spike ELISA, S/T value was calculated by dividing the OD for 1:100 dilution by 0.4; and, for Architect the system calculates a calibrator mean chemiluminescent signal and the default result unit is index (S/C). Cut-off value was 1 and 1.4 for Spike ELISA and the Architect, respectively. The 105 samples were differentiated into: concordant positive (n = 40), concordant negative (n = 58) and discordant (n = 7).

Signals for both the assays were plotted for all 105 serum samples (Fig 1). The IgG S/T value of the CDC ELISA for the seven discordant samples ranged from 1.5 (weak positive) to 6.5 (strong positive), with a mean of 3.4 and 0.45 coefficient of variance. Among the serum samples with concordant positive results the mean IgG S/T value of the CDC ELISA was 5.7 and the mean S/C value of the Architect assay was 6.1.

**Fig 2:**
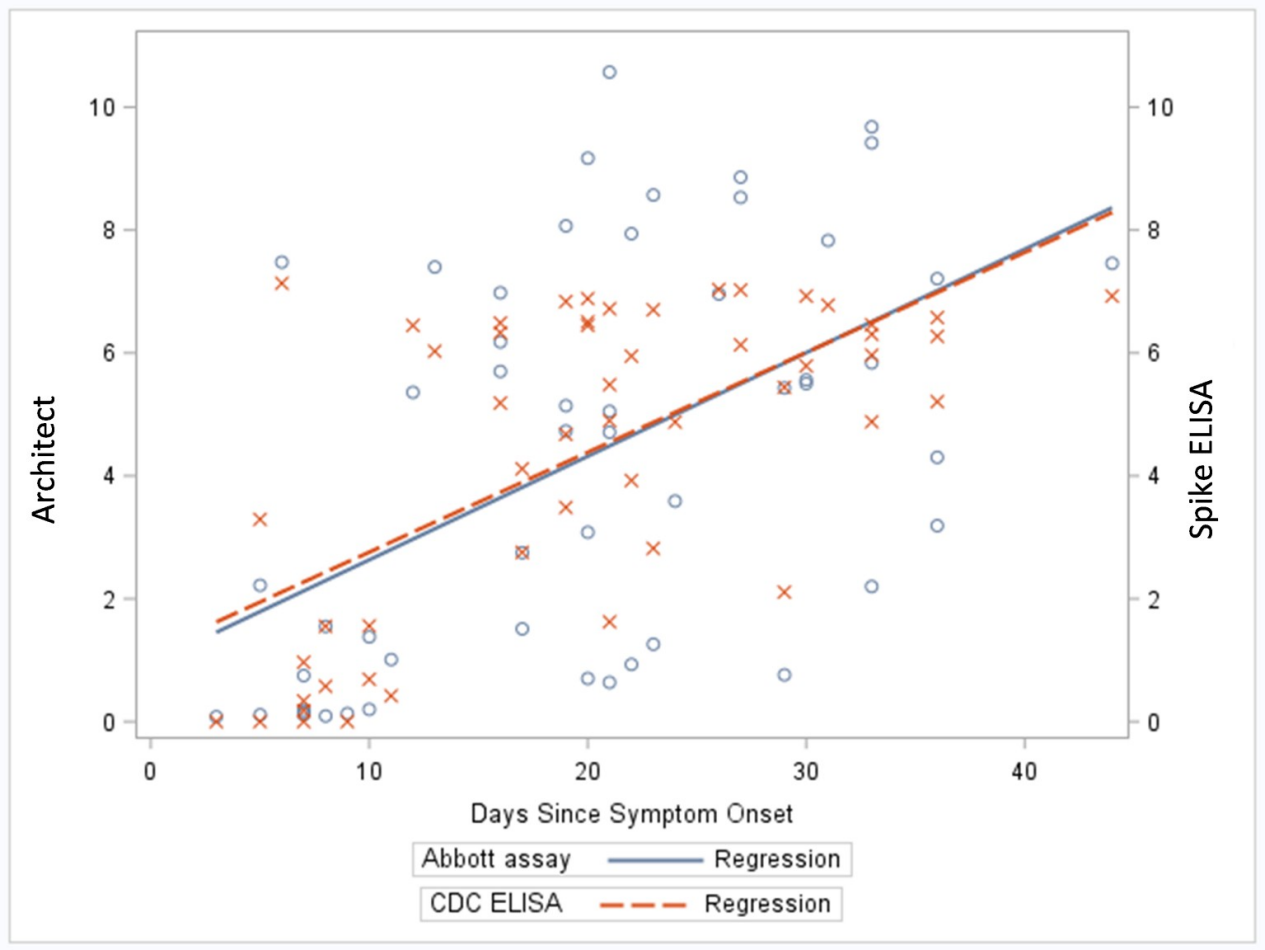
Comparison of SARS-CoV-2 spike protein ELISA (spike ELISA) and Abbott Architect^TM^ SARS-CoV-2 IgG assay using serum samples from individuals with a positive rRT-PCR test result (n = 52) by days since symptom onset. *Each blue circle (O) represents a serum sample tested by the Architect assay; each red cross (X) represents a serum sample tested by the Spike ELISA. Samples collected from asymptomatic individuals (n = 4) were excluded from the analysis.

Among serum samples from individuals with positive rRT-PCR results, IgG signal increased with days since symptom onset for both the anti-nucleocapsid protein and anti-s protein-based assays (Fig 2). Sensitivity of the two assays is influenced by time since symptom onset and time since exposure (data not shown).

## Discussion

We compared side-by-side results from two serologic assays, one that detects antibodies to the S- protein and one that detects antibodies to the N-protein of SARS-CoV-2 virus. Our results showed a concurrence of 93.3%, suggesting strong agreement between the two assays when used in patients to detect a history of infection with SARS-CoV-2.

In this study, all discordant samples (n = 7) were positive by the spike-protein-based assay (Spike ELISA) and were negative by the nucleocapsid-based immunoassay (Architect). Our findings are similar to a recent study conducted by Liu et al., where an spike-protein-based ELISA was found to be more sensitive than an nucleocapsid-based assay [14]. The predetermined quantitative threshold of a qualitative positive test as identified by the manufacturer might also vary from one assay to another. The discordance could therefore be attributed to inherent variations in assay design, target antigens, or expected differences in individuals’ polyclonal antibody responses in the timing of specific antibodies generated from infection [15].

Studies have shown that seroconversion for IgG occurs earlier for SARS-CoV-2 anti-N antibodies than for anti-S antibodies and IgG reactivity against SARS-CoV-2 N antigen was detectable by 2 weeks after symptoms onset [16–18]. This is similar to data from SARS-CoV-1 and other human coronavirus infections, in which antibodies against the N-protein were detected significantly earlier than antibodies to the S-protein [19]. Nucleocapsid (N) is produced in abundance during the early phases of viral replication [20]. If viral replication is not controlled well by the innate or early T cell immune responses, N could be abundant and therefore induce a robust antibody response [21]. However, if replication is well controlled and the immune system primarily sees intact virions, the antibody response might be spike dominant. These findings demonstrate that serologic assays face limitations inherent to the human immunologic response, and may therefore provide some discrepant results even when testing the same samples across different assays [22].

Further studies are needed to understand how patient and illness characteristics may result in variability among immunoassay performance, including shortly after infection (<14 days after symptom onset). Given the overall sample size and small number of discordant results, this analysis lacked statistical power to evaluate these characteristics by assay performance. It has been proposed that serologic testing could be reliably used after 14 days post symptom onset [23], since sensitivity within the first 14 days of symptom onset is highly variable for most SARS-CoV2 antibody assays but improves after 14 days [9, 24]. Analyses on serologic assay performance using sera collected <14 days of symptom onset provide a clearer picture of assay performance during the early phase of disease.

Currently, in the United States, the three authorized COVID-19 vaccines are based on the SARS-CoV-2 spike protein [25]. As vaccines become universally available, differentiating SARS-CoV-2 antibodies induced by vaccination versus natural infection may provide valuable epidemiologic information. Multiplex serology testing developed to measure antibody response to more than one antigen might be useful to monitor asymptomatic infection rates and assess for reinfection, vaccine breakthrough, and population-level immunity achieved by either natural infection or vaccination. Because current US-authorized vaccines utilize S protein antigens only, serology tests that assess spike protein antibodies can be used to evaluate immune response to vaccination, whereas those targeting nucleocapsid protein antibodies could serve as markers for natural infection.

In conclusion, our data indicates that both the spike ELISA and Architect assay had high agreement, but rarely paired tests results can vary when using two different serologic assays. Choosing a serologic assay requires consideration of current and past incidence of COVID-19 in a geographic area, characteristics of the targeted patient population to be tested, vaccination history and required performance characteristics of the test.

## Supporting information

S1 Data (XLSX)
